# E-cigarette use and its predictors: Results from an online cross-sectional survey in Poland

**DOI:** 10.18332/tid/113093

**Published:** 2019-11-08

**Authors:** Pawel Lewek, Beata Woźniak, Paulina Maludzińska, Janusz Smigielski, Przemyslaw Kardas

**Affiliations:** 1 Department of Family Medicine, Medical University of Lodz, Lodz, Poland; 2 Nowy Dwor Medical Centre, Nowy Dwor, Poland; 3 J. Babinski Psychiatric Hospital, Lodz, Poland; 4 Social and Technical Department, State Higher Vocational School, Konin, Poland

**Keywords:** smoking cessation, electronic nicotine delivery systems, vaping, surveys and questionnaires, e-cigarettes

## Abstract

**INTRODUCTION:**

Since the invention of electronic cigarettes (ECs) in 2003, their use has spread worldwide; however, little is known about the profiles of EC users. Understanding the motivators for using ECs enables more accurate prediction of their use and more effective direction of pro-health activities. Our objective was to identify the factors that may influence the decision to use ECs and their possible adverse effects according to the experiences of EC users.

**METHODS:**

A cross-sectional online survey was administered between 1 July 2016 and 1 January 2017 among 1288 Polish-speaking users of social networks and EC forums. To explore associations between current EC use and other factors, multivariate binary logistic regression analyses were performed.

**RESULTS:**

The final analysis included 1142 survey participants: mean age 25.9 years (± 11.1), 85.6% were male, 50.3% had secondary education, 98.2% were Polish citizens, and 81.0% were current EC users. Male gender, lower education, aged ≤40 years, former cigarette smoking, previous attempts to quit smoking, perception of lack of harmful effects of ECs, perception of ECs as being tastier and cheaper than cigarettes, awareness of the advantages of ECs and their use as a smoking cessation aid were all statistically significant factors increasing the risk of EC use. The majority of study participants claimed that ECs are less addictive or not addictive compared to cigarettes (62.6%) and less harmful or not harmful (89.5%) compared to cigarettes. The most common reported side effects of ECs were dryness in the mouth (8.3%), itching in the throat (4.5%) and nausea (1.9%).

**CONCLUSIONS:**

Males aged ≤40 years with a lower level education were more likely to use ECs in the studied Polish population. The perception that ECs are less harmful than regular cigarettes is a factor increasing the odds of EC use; however, although ECs have few adverse effects, they nevertheless exist.

## INTRODUCTION

The electronic cigarettes, or e-cigarettes (ECs), are hand-size battery-operated devices designed to resemble a traditional cigarette, usually used to inhale nicotine-containing vapour^1^. Puffing ECs activates a battery-operated heating element in the atomizer and liquid, which consists of propylene glycol, glycerine, nicotine, tobacco extracts, adulterants and/ or flavourings^2^. It is well known that the burning of tobacco in cigarettes leads to the production of almost 4000 compounds, with more than 60 classified as carcinogens^3^. Instead of toxic smoke, ECs produce vapour from heating a liquid, a process that is believed to be less harmful to the user, nonetheless toxic and carcinogenic carbonyl compounds have also been found in EC vapour^4^. E-cigarettes were reportedly designed initially as a healthier alternative to cigarettes by an inventor whose father died of tobacco-smokerelated lung cancer^5^. The device was first introduced to the Chinese domestic market in 2003 as an aid for smoking cessation and a replacement for regular cigarettes^1^.

Since then, ECs have gained popularity around the world. They are mostly promoted via the Internet but recently also by the entertainment industry. E-cigarettes are widely available through online stores or from retail outlets such as small kiosks in shopping malls or petrol stations^6^.

Although the sales of ECs increased from an estimated $11.6 million in 2010 to $751.2 million in 2016 in the US^7^, and up to $10 billion globally in 2017^8^, there is growing concern about their use among smokers due to limited scientific evidence concerning their safety and efficacy^9^. E-cigarette use has rapidly displaced the tobacco products market around the globe. As Poland is one of the seven largest e-cigarette markets worldwide^10^, the Polish population is an important group for studies of e-cigarette use. The present study attempts to identify predictors that may be used to indicate the factors that are most likely to motivate people to use ECs.

Most of the current research in the field of electronic cigarettes is devoted to the chemicals used in them and their effect on health. Few studies examine the possible factors affecting the use of ECs. A study by Penzes et al.^11^ on 826 Hungarian smokers found the major motivators for using ECs to be curiosity/taste variety (positive) and fear of danger of dependence (negative); however, the study was limited to university students and included only 206 e-cigarette users^11^. Similarly, a Spanish study by Bunch et al.^12^ (n=600) indicated that the most important drivers were a desire to reduce tobacco smoking and the will to quit or to use ECs in places where smoking is prohibited. In Poland, Brożek et al.^13^ concluded that the leading motivators were: quit tobacco use, reduce the impact on health, and reduce costs. However, the study was conducted also on students^13^. While other studies have also examined the topic, the subjects were mainly adolescents or students^14,15^.

Few studies have examined the predictors of e-cigarette use in Central and Eastern Europe, which in fact are crucial to understanding the phenomenon of e-cigarette use and to determine the motivational factors associated with electronic cigarette use. By being able to predict who is more likely to use ECs, preventive actions may be more accurately targeted at specific groups of people who are more likely to start using them. Hence, the aim of this study was to explore the possible predictors that encourage electronic cigarette use within the Polish market with one of the largest national populations of e-cigarette users. In addition, it explores the most common positive health impacts and adverse effects of electronic cigarette use.

## METHODS

### Participants and procedure

A cross-sectional online survey was performed among Polish-speaking individuals to collect information from people familiar with e-cigarettes. The survey questionnaire was designed based on a review of available literature and the authors’ experience. The survey was made available in Polish through a web-based surveying platform (Survey Monkey) between 1 July 2016 and 1 January 2017. The invitation to the survey was distributed across social networks including related Facebook groups and internet forums devoted to e-cigarette use (Supplementary file, Document 1). The link to the survey was not password-protected and hence was open to anyone who was willing to complete it. However, the option to complete the questionnaire twice was disabled: the initial question asked about previous participation in this survey – if confirmed, the participant was not able to continue. The survey was completely anonymous, IP numbers were not collected.

To be included in the final analysis, the participants had to meet the following criteria: no previous participation in the survey, aged 13–70 years, an answer provided to at least one question other than just the demographic questions, and awareness of e-cigarettes.

In total, 1288 responses were collected. Of these, 146 questionnaires did not meet the inclusion criteria and were excluded from the analysis, these included: 16 duplicates, 86 without the age provided, 6 with age outside the inclusion range, 35 with only demographics provided, 2 did not answer the question about knowledge of e-cigarettes, and one denied such knowledge. As a result, 1142 responses were analysed. As it was possible for a participant to skip questions, the total number of respondents might vary in some questions. If so, the number (N) of participants was given when a particular result was presented.

### Measures

On entering the survey participants were asked: ‘Have you ever filled in this questionnaire prepared by the Department of Family Medicine, Medical University of Lodz, regarding e-cigarettes?’. Only those who answered ‘no’ were allowed to continue, those who answered ‘yes’ were not and were counted as ‘duplicates’ and their answers were excluded from final analysis. Next, they were asked about age, sex, education, country of residence, and place of residence. Participants were divided into four groups according to age: students of Primary and Secondary school (aged <18 years), university students (18–25 years), young adults (26–45 years) and middle-aged adults (>45 years). Next question was: ‘Have you ever heard about electronic cigarette named also an e-cigarette?’ – participants who answered ‘no’ were excluded from the study, participants who answered ‘yes’ were able to continue. Participants who answered ‘yes’ to the question ‘Do you vape e-cigarettes currently?’ were categorized as ‘current e-cigarette users’. Those who denied doing so, but answered ‘yes’ to the question ‘Have you been vaping e-cigarettes in the past?’ were categorized as ‘former EC users’. No participant indicated occasional e-cigarette use, thus ‘current EC use’ meant also ‘daily EC use’. In the same way, respondents who answered ‘yes’ to the question ‘Do you smoke regular cigarettes currently?’ were categorized as ‘current cigarette smokers’. Those who denied doing so, but answered ‘yes’ to the question ‘Have you been smoking regular cigarettes in the past?’ were categorized as ‘former cigarette smokers’. No participant indicated occasional use of regular cigarettes, thus ‘current cigarette smoking’ meant also ‘daily smoking’. Participants who were ‘current e-cigarette users’ and ‘current cigarette smokers’ were categorized as ‘dual-users’. Participants who were ‘former e-cigarette users’ and ‘former cigarette smokers’ were categorized as ‘former dual-users’, i.e. ‘quitters’. In this paper, ‘current e-cigarette users’ is interpreted as all e-cigarette users, irrespective if they smoked regular cigarettes or not (i.e. ‘current e-cigarette users’ include ‘dual-users’). The same with ‘current smokers’ – this group includes all regular cigarette smokers, irrespective if they used ECs or not. By ‘current only e-cigarette users’ we mean participants who used only ECs (i.e. ‘dual-users’ are excluded from this group) and by ‘current only cigarettes smokers’ we mean participants who used only regular cigarettes (i.e. ‘dual-users’ are excluded from this group).

Current e-cigarette users were asked ‘What nicotine concentration do you use now?’ and were allowed to type in three concentrations (mg/mL). Those who answered with a zero were classified as EC users of liquid without nicotine, while those who answered with a ≥1 were classified as users of liquid with nicotine. If participants filled in a zero in any of three fields then they were classified as EC users of liquids with and without nicotine. Answers to the question ‘What adverse effects have you experienced while using e-cigarettes?’ were used to list the most common side effects of e-cigarette use.

The survey was piloted in a group of 20 volunteers and fine-tuned according to the feedback obtained. The final version of the questionnaire comprised 38 questions, most of which offered single-choice responses on a 4-point Likert scale and a ‘difficult to say’ option.

### Ethical approval

Ethical approval was not necessary according to the Ethics Committee, Medical University of Lodz, as this was not an experimental study.

### Statistical analysis

The data were verified for normality of distribution and equality of variances. The results of the quantitative variables were presented as mean (± SD), median, minimum and maximum. Chi-squared tests were performed to compare nominal variables and to examine the univariate associations between sociocultural factors and e-cigarette use. Fisher’s test was used for values below 3; the chi-squared test with Yates correction was used for values below 6.

Multivariate logistic regression analysis was performed to identify correlates of current e-cigarette use. Independent variables included demographics, as well as all variables identified by univariate analysis to have a statistically significant association with e-cigarette use, with an odds ratio calculation. The level of statistical significance was set at p<0.05 for all the analyses. The statistical analysis was performed with Statistica version 13 (Statsoft Polska).

Of all the survey questions, only answers to selected queries are presented in this paper – the choice was made on the basis of topic and statistical significance (the data supporting the findings of this study are available from the corresponding author upon reasonable request).

## RESULTS

Most of the study participants were male (85.6%), most had secondary or higher education (74.8%), lived in Poland (98.2%) and were residents of urban areas (72.1%). More detailed characteristics are presented in Table 1.

**Table 1:** Characteristics of the participants included in the final analysis (N=1142)

	*n (%)*
**Age** (years)	
<18	134 (11.7)
18–25	603 (52.8)
26–45	312 (27.3)
>45	93 (8.1)
minimum	13
maximum	69
mean (± SD)	25.9 (± 11.1)
median	21
**Gender**	
Male	978 (85.6)
Female	164 (14.4)
**Education**	
Higher	280 (24.5)
Secondary	575 (50.3)
Vocational	134 (11.7)
Primary	153 (13.4)
**Country of residence**	
Poland	1121 (98.2)
Other	21 (1.8)
**Place of residence**	
City >1 M	140 (12.3)
City 0.5–1 M	134 (11.7)
City 0.1–0.5 M	225 (19.7)
City 10–100 K	322 (28.2)
City <10 K	119 (10.4)
Rural area	202 (17.7)
**E-cigarettes users**	
Current	925 (81.0)
Former	45 (3.9)
Never	81 (7.1)
No data	91 (8.0)
**Cigarettes smokers**	
Current	134 (11.7)
Former	704 (61.6)
Never	133 (11.6)
No data	171 (15.0)
**Liquids used by EC users**	
Only with nicotine	655 (57.4)
Only without nicotine	50 (4.4)
With and without nicotine	177 (15.5)
No data	260 (22.8)

M: million inhabitants, K: thousand inhabitants.

Participants were well aware of ECs – only three persons out of 1145 (0.3%) were not. The majority (925; 81.0%) of participants declared themselves to be current e-cigarette users, while only 45 (3.9%) claimed to have used electronic cigarettes in the past. In total, 970 (84.9%) participants were recognized as current or ever e-cigarette users, while 81 (7.1%) reported never using ECs.

Regarding cigarette smoking: 134 (11.7%) participants reported current cigarette use, 704 (61.6%) were former smokers, and 133 (11.6%) had never smoked. In addition, 171 (15.0%) persons did not answer either question. In total, 838 (73.4%) of the participants were ever cigarette users.

More than half (655; 57.4%) of current e-cigarette users used liquids with nicotine, 177 (15.5%) used liquids with and without nicotine, and only 50 (4.4%) reported using only liquid without nicotine.

Of the 810 respondents who answered questions about current (or former) e-cigarette use (or smoking), 109 (13.1%) declared themselves to be dual-users (i.e. current e-cigarette users and cigarette smokers), 33 (3.9%) claimed to use both ECs and cigarettes in the past (former EC users and former cigarette smokers, i.e. former dual-users, quitters), and 665 (79.5%) were current e-cigarette users and former smokers. Current smoking and former EC use were declared by 29 participants (3.5%).

Most EC users (57.4%) used liquids with nicotine; however, some used also liquids without nicotine (15.5%) or both (15.5%) (Table 1).

Mean age of current EC users was 25.5 years (range 13–69), median 20. The average age of former e-cigarette users was 31.4 (14–65), median 27. The average age of current smokers was 22.4 years (13–60), median 19, for former smokers 27.3 years (13–69), median 22, and for current dual-users 25.5 years (13–54), median 19. There was only one former dual-user (i.e. quitter), aged 17 years. The average age of current only e-cigarette users was 26.1 years (13–69), median 21. The average age of current only smokers was 27.0 years (16–60), median 25.

The majority of respondents had a favourable opinion of electronic cigarettes. Most (62.6%) declared that ECs were less addictive than regular cigarettes or not addictive at all. About one fourth (29.2%) thought that they were equally or more addictive. The majority of respondents (89.5%) reported that ECs were less harmful than regular cigarettes or not harmful at all and that they experienced positive effects from e-cigarette use (91.8%) (Table 2). This positive image of ECs was reinforced by personal experience, as most participants (66.1%, 755 in total; 63.8%, 729 current users and 2.3%, 26 former users) did not report experiencing any side effects. Only 12.1% (138) participants had this kind of experience (11.1%, 127 current users, and 1.0%, 11 former users).

**Table 2:** Participant opinions on EC addictiveness, harmfulness and the positive effects of EC use (N=1142 )

	*n (%)*
**Do you think that e-cigarettes are:**	
More than or equally addictive as regular cigarettes	332 (29.2)
Less addictive than regular cigarettes or not addictive at all	716 (62.6)
Difficult to say	81 (7.1)
No data	13 (1.1)
**Do you think that e-cigarettes are:**	
More than or equally harmful as regular cigarettes	61 (5.3)
Less harmful than regular cigarettes or not harmful at all	1022 (89.5)
Difficult to say	42 (3.7)
No data	17 (1.5)
**Have you experienced any positive effects of using e-cigarettes?**	
Yes	1048 (91.8)
No	52 (4.5)
No data	42 (3.7)

Most of the respondents (66.9%, 764) reported being introduced to nicotine intake through regular cigarettes, while one in ten (10.1%, 115) started using nicotine with electronic cigarettes. Only 1.1% (12) of respondents reported using other products like snuff or snus as initial nicotine products, and 1.1% (14) responded ‘hard to say’.

Nevertheless, respondents experienced some adverse effects. The prevalence of these effects was, however, not very high: dryness in the mouth, itching in the throat, and nausea were adverse effects realized by a minority of respondents (8.3%, 4.5%, 1.9%, respectively, Table 3.

**Table 3:** Adverse effects of e-cigarettes reported by current and former EC users

	*Total (n=1032 ) n (%)*	*Current users (n=907 ) n (%)*	*Former users (n=125 ) n (%)*
Dryness in mouth	86 (8.3)	80 (8.8)	6 (13.3)*
Itching in throat	46 (4.5)	40 (4.4)	6 (13.3)*
Nausea	20 (1.9)	19 (2.1)	1 (2.22)

* p<0.005, based on chi-squared test.

It might be hypothesised that some adverse effects lead to discontinuation of use: dryness in the mouth and itching in the throat were associated with former use (p<0.005). No correlation was found between nausea and use (Table 3). None of the three adverse effects was associated with current use (p>0.05) (Table 3).

The respondents most commonly listed a lack of unpleasant odour (82.8%), improved smell sensation (65.0%) and improved taste sensation (63.0%) as advantages of ECs over regular cigarettes (Table 4).

**Table 4:** Advantages of e-cigarettes over regular cigarettes reported by respondents (N=1142)

*Advantages*	*n (%)*
Lack unpleasant odour	946 (82.8)
Improved smell sensation	742 (65.0)
Improved taste sensation	718 (63.0)
Cough reduction	698 (61.1)
Easier breathing	741 (64.9)
Saving money	818 (71.6)
Other	143 (12.5)

Predictors of e-cigarette use were established by univariate and multivariate analyses. Univariate analysis found use to be highly associated with age ≤40 years, male gender and primary or secondary education. Other factors were found to affect the chance of using e-cigarettes, such as: the belief that e-cigarettes were less harmful than regular cigarettes (increased), perception of ECs as more fashionable than regular cigarettes (increased), knowledge that ECs were cheaper than smoking (increased), seeing the advantages of using ECs (increased), declaration of quitting smoking as a significant motivator for using ECs (increased), using ECs as a device to help quit smoking (increased), quitting smoking for more than six months (decreased) (Table 5).

**Table 5:** Factors identified in univariate and multivariate regression analysis models affecting current EC use (dependent variable)

*Covariates*	*Univariate analysis*		*Multivariate analysis*	
	*OR ( 95% CI)*	*p*	*OR ( 95% CI)*	*p*
**Age** (years)				
>40	Ref.	-	Ref.	-
≤40	1.68 (1.02–2.79)	<0.05	1.99 (1.34–2.94)	<0.001
**Gender**				
Female	Ref.	-	Ref.	-
Male	8.11 (5.37–12.27)	<0.001	5.87 (3.80–9.04)	<0.001
**Education**				
Higher	Ref.	-	Ref.	-
Primary or Secondary	3.47 (2.53–4.77)	<0.001	2.46 (1.72–3.51)	<0.001
**Place of living**				
Rural	Ref.	-		
City	0.56 (0.31–1.01)	0.053	-	-
**Possibility of using substances other than nicotine liquids**				
No	Ref.	-	Ref.	-
Yes	8.31 (2.02–34.16)	<0.005	4.83 (1.15–20.19)	<0.05
**Current cigarette smoker**				
No	Ref.	-	Ref.	-
Yes	0.56 (0.34–0.91)	<0.05	0.51 (0.29–0.90)	<0.05
**Former cigarette smoker**				
No	Ref.	-	Ref.	-
Yes	11.54 (7.16–18.59)	<0.001	18.97 (10.24–35.14)	<0.001
**Starting to use e-cigarettes in order to quit smoking**				
No	Ref.	-	Ref.	-
Yes	4.69 (2.90–7.57)	<0.001	2.03 (1.11–3.72)	<0.05
**Starting to use e-cigarettes because they were less harmful than cigarettes**				
No	Ref.	-	Ref.	-
Yes	13.20 (8.34–20.91)	<0.001	3.04 (1.60–5.79)	<0.001
**Starting to use e-cigarettes because they were more fashionable**				
No	Ref.			
Yes	2.73 (0.98–7.60)	0.06	-	-
**Starting to use e-cigarettes because using them is cheaper than smoking cigarettes**				
No	Ref.	-	Ref.	-
Yes	12.44 (7.28–21.40)	<0.001	2.80 (1.44–5.45)	<0.005
**Sees advantages of using ECs**				
No	Ref.	-	Ref.	-
Yes	37.71 (18.12–78.50)	<0.001	21.97 (8.39–57.47)	<0.001
**Used e-cigarettes as an aid to quit smoking**				
No	Ref.	-	Ref.	-
Yes	7.76 (4.87–12.36)	<0.001	5.31 (3.19–8.81)	<0.001
**Has quit smoking for more than 6 months**				
no	Ref.			
yes	0.80 (0.43–1.50)	0.48	-	-

The significant independent variables identified in univariate analysis were then analysed in multivariate regression analysis. Multivariate regression models found 11 out of 14 variables to correlate with current use; only place of living, former smoking of cigarettes, use of ECs because they are fashionable, and quitting smoking for more than six months were not associated with current use. Full results of the logistic regression are presented in Table 5.

## DISCUSSION

In this study we have found some important associations. First, age ≤40 years, male gender and primary or secondary education were found to be associated with current use. Other identified factors affecting current use were: using substances other than nicotine liquids, current or former cigarette smoking, starting ECs in order to quit smoking, starting e-cigarettes because they were less harmful, more fashionable or cheaper than regular cigarettes, seeing advantages of ECs and using them as a method of smoking cessation. Second, we found that most respondents claimed that ECs are less addictive and less harmful than regular cigarettes. Third, dryness in mouth and itching in throat were reported as statistically significant adverse effects of e-cigarette use among former users.

In our study, participants aged ≤40 years were twice as likely to use ECs as those >40 years. Association of age ≤40 years with e-cigarette use was also confirmed in other studies^16,17^, especially in young adults across the European Union. In an analysis based on the Eurobarometer 385 survey on 26566 participants^17^, it was found that respondents aged 15–24 years were 3.3 times and those aged 25–39 years were 1.89 times more likely to have used ECs, which is comparable to our results. However, while no significant difference was found between the sexes in that study, a strong association of use was observed with males in the present study (OR=5.87). It could be that our studied population was actively using ECs. Vardavas et al.^17^ report that 20.3% of respondents reported having ever used ECs compared to 84.9% in the present study^17^.

Males were found to have almost six times higher odds of using ECs than females, and those with only primary or secondary education had almost three times higher odds of becoming e-cigarette users than those with higher education. Although previous studies have also noted a similar trend with regard to gender^18^, the present study is the first to note such a strong correlation between gender/education and e-cigarette use among the Polish population. Probably, mainly primary or secondary male students are reaching for ECs. This correlation is well confirmed as a pattern of e-cigarette use in the US population^19^.

In a study by Harlow et al.^20^ lower education was associated with reduced odds of exclusive e-cigarette use and cigarette cessation without e-cigarettes, which may suggest differences between US and Polish populations. In contrast, lower education is presented as a favourable factor for e-cigarette use in a US Department of Health and Human Services 2016 report^19^.

Participants who were aware of the possibility of inhaling chemicals, other than nicotine, within the vapour had almost five times higher odds in identifying themselves as e-cigarette users than participants who did not, which may suggest that using substances other than nicotine may be a strong incentive to start using ECs. One such example is the selection of flavour compounds, which were found by Penzes et al.^11^ to be a major motivator for using ECs^11^. Initiation in and quick progress to tobacco smoking is associated with higher risk of becoming a chronic smoker later in life^21^, while exposure to nicotine at a developmental age is associated with an increased risk of mood and attention irregularity symptoms^22^. In the present study, one in ten respondents was first exposed to nicotine through ECs; it is possible that these e-cigarette users may switch to regular cigarettes in the future^9^. It is well known that e-cigarette use is strongly associated with the use of other tobacco products, especially among teenagers and young adults^19^. Our findings suggest that being a former cigarette smoker increases the odds almost 19-times of current e-cigarette use.

Twenty-five per cent of Europeans reported using ECs because they were cheaper than regular cigarettes^23^. The cost was an important factor in the decision to begin using e-cigarettes, confirmed also in our study; participants claiming that ECs were less costly were more than three times more likely to use ECs. It is possible that aligning ECs and cigarette prices may be an option to limit their recreational use.

Many studies, including the present study, found that e-cigarette users perceived ECs as being less harmful to health than regular cigarettes^24^. Depending on the study, between 17% and 82% of participants perceive ECs as being less harmful than cigarettes^25^, this rose to 89.5% in the present study. The belief that ECs are harmless is one of the most often cited (among curiosity and flavour/taste) reasons for starting e-cigarette use^19^. As such, unsubstantiated belief in the positive health effects of EC use may be harmful for users; specific regulations for media information about ECs are of high importance. Currently, Polish regulations follow the EU Directive on tobacco products^26^ and do not allow the sale of e-cigarettes to people younger than 18 years. E-cigarettes cannot be used in public places where regular cigarettes are forbidden. Their sale is not allowed in vending machines, healthcare facilities and across the border. They cannot be advertised, and neither ECs nor e-liquids may be promoted. According to current regulations EC packaging should include warnings, such as: ‘This product contains nicotine which is a highly addictive substance’, or ‘This product contains nicotine which is a highly addictive substance. It is not recommended for use by non-smokers’^27^.

This perception that ECs are harmless could arise from positive media coverage. Television has been found to previously be a major source of information about ECs^28^, being most commonly advertised as a healthier alternative to regular cigarettes^29^. A Eurobarometer report found that 55% of Europeans (56% of Poles) believed ECs to be harmful to health, with this proportion showing a 3% increase in the last three years^30^. Such beliefs represent an important factor in deciding to use ECs; e.g. our present findings indicate that participants who thought ECs were less harmful than cigarettes were three times more likely to use them. This is consistent with a recent Australian study in which nicotine vaping product (NVP) users were almost four times more likely to perceive NVPs as less harmful compared to combustible cigarettes than NVP non-users^31^. Similar results were confirmed in a survey among Texas youth (students in grades 6, 8 and 10) in which current EC users had almost five times greater odds of reporting that ECs were ‘not at all’ harmful compared to non-current e-cigarette users, while e-cigarette users were almost four times more likely than never users^32^. A 2013 British study found respondents aged 11–18 years were almost twice as likely to claim that ECs are less harmful than cigarettes if they tried ECs or used them sometimes^25^. Lower perceived harm of e-cigarettes is associated with higher odds of openness to and curiosity about trying ECs^33^ and an increased likelihood of trying e-cigarettes, especially among adolescents^34^.

One in eight (15.5%) of our respondents claimed to have experienced adverse effects from e-cigarette use, which indicates that ECs may present some harmful effects. The most common adverse effect reported was dryness in the mouth (8.3%). Dryness was also found to be the most common symptom (38.9%) in an international study that included Polish respondents^24^, of whom 57.9% reported various adverse effects. This was a much higher percentage than that found in the present study; unfortunately, separate data were not given for the Polish respondents. Dryness in mouth and throat was also the second major adverse effect reported by 3.7% of the participants among 1042 Hungarian users^35^. The adverse symptom in that study was coughing (4.0% of participants), which was not one of the main symptoms reported by users in our study. In the present study, a strong correlation was found between being a former e-cigarette user and either dryness in the mouth or an itching sensation in the throat (p<0.005), which suggests that adverse effects may be a key motivator for quitting e-cigarette use. Our findings are consistent with other publications confirming mouth or throat irritation and nausea to be adverse effects of e-cigarette use^36^. However, in contrast to other studies, the participants did not report headaches or dry cough due to e-cigarette use^37^.

Due to the sample choice in our study, the studied population was well aware of ECs. The link to the survey was distributed on e-cigarette user groups and forums, which typically possess strong knowledge of ECs. Social media and internet forums usually gather people interested in one specific topic; so EC forums were targeted in our study, leading to 99.7% (1145) of this study’s participants being aware of ECs. Overall awareness of ECs in other studies, both European and US, was high and usually exceeded 80%^17,24,38^.

Current cigarette smoking was not strongly associated with e-cigarette use as was past cigarette smoking. People who reported currently smoking had twice lower odds (OR=0.51) of using ECs than those who did not. On the other hand, former smokers had 20-times higher odds (OR=18.97) of using ECs than non-smokers. This supports the notion that ECs may be effectively used as a healthier substitute for regular cigarettes; ECs are most commonly used by ex-smokers who quit regular cigarettes after switching to ECs. This result is consistent with ECs being used as a smoking reduction or anti-tobacco craving aid reported in many studies^36^. According to the Eurobarometer survey 2017, smokers are the primary users of ECs, with only 3% of Polish (4% of EU) never smokers ever trying e-cigarettes^30^.

On the other hand, almost every participant (91.8%,1142) reported experiencing positive health effects from e-cigarette use, with the lack of unpleasant odour, improved smell and taste being the most common, consistent with other studies^24^. It is worth noting that participants who declared that e-cigarette use had some advantages had 22-times higher odds to use ECs than those who did not. Knowledge of these positive effects strongly correlates with commencement of e-cigarette use.

Survey participants who used ECs to quit smoking had more than five times higher odds of using ECs, suggesting that e-cigarettes were used more by smokers than non-smokers. This was consistent with a pan-European Eurobarometer survey where most of participants (61%) reported using ECs to reduce tobacco consumption^30^. However, a recent meta-analysis on the basis of 20 studies reported that smokers who use ECs have 28% lower odds of quitting smoking than those who do not^39^. Our results suggest that smokers may be misled and use ECs believing that they will help them quit smoking, while in fact they only reinforce their habit.

### Limitations and strengths

The obvious limitation of our study was the selection bias present in the recruitment of subjects. Due to the way the invitation to the study was distributed, the study group was composed of a high proportion of e-cigarette users. Moreover, the target population was limited to the users of internet social media and forums; those who do not use the internet were not reached by our study. This, however, would not have resulted in a significant bias: according to recent data, as many as 81.9% of Polish households have access to Internet^40^. Hence, an online survey seems to be one of the best ways to reach the targeted population.

It could be argued that the results of the study reflect primarily the opinions of young Polish males with secondary education, as this group was the most numerous among the respondents. However, a similar dominance of male users has also been observed in other studies^24^. Although our results cannot be generalised for Polish or European populations, the high number of respondents (over 1100) nevertheless gives a novel insight into predictors of e-cigarette use among current users and allows for better understanding of this behaviour among Polish e-cigarette users.

## CONCLUSIONS

Age up to 40 years, male gender and lower education were found to have higher odds of current e-cigarette use in a Polish population. Other identified factors affecting current e-cigarette use include the possibility of using substances other than nicotine liquids, current or former cigarette smoking, starting ECs to quit smoking, starting ECs because they were less harmful or cheaper than regular cigarettes, perceiving ECs to have advantages over cigarettes, and using e-cigarettes as a method to quit smoking. Despite having possible adverse effects, ECs are widely believed to be less harmful and less addictive than regular cigarettes, which may lead to unsubstantiated belief that they constitute a harmless nicotine-delivering device. There is still a strong need to study electronic cigarettes and understand why users choose them over other nicotine-delivering devices.
