# Supplementary material for: E-cigarette use and its predictors: Results from an online cross-sectional survey in Poland
Source: Tob Induc Dis. 2019 Nov 8;17:79. doi: 10.18332/tid/113093 (PMC6856825; doi:10.18332/tid/113093)
Supplement: Supplementary file 1 [file TID-17-79-s1.pdf]

Supplementary file, Document 1

Name of Polish E-cigarettes Facebook groups where the survey invitation was distributed:

E - Papieros Polska V2
Zachmurzeni - giełda / recenzje / nowinki.
E-Papieros POLSKA
E-PAPIEROS Sprzedam - Kupię - Wymienię.

Polish Internet forums devoted to EC usage where the survey invitation was distributed:

http://forum.e-palarnia.com/

http://www.swiatpapierosow.pl/

http://forum.vape.pl/index.php

http://e-papierosy-forum.pl/

© 2019 Lewek P.
